# Class I malocclusion with severe double rotrusion treated with first premolars extraction^*^

**DOI:** 10.1590/2176-9451.19.3.127-138.bbo

**Published:** 2014

**Authors:** Ricardo Moresca

**Affiliations:** 1 PhD in Orthodontics, School of Dentistry - University of São Paulo. MSc in Orthodontics, Methodist University of São Paulo (UMESP). Specialist in Orthodontics, Federal University of Paraná (UFPR). Adjunct professor, Department of Orthodontics, Federal University of Paraná (UFPR). Full Professor and Head of the Postgraduate program in Orthodontics, Positivo University. Certified by the Brazilian Board of Orthodontics and Facial Orthopedics (BBO).

**Keywords:** Angle Class I malocclusion, Orthodontic space closure, Orthodontic anchorage procedures

## Abstract

Angle Class I malocclusion with bimaxillary protrusion is characterized by severe buccal tipping of incisors, which causes upper and lower lip protrusion. First premolars extraction is recommended to reduce facial convexity as a result of anterior teeth retraction, which keeps canines and first molars in key to occlusion. In order to yield orthodontic results that are compatible with ideal esthetic and cephalometric outcomes, the space closure phase needs to be carried out with overbite and incisors torque control. The majority of cases also requires maximum anchorage of posterior teeth. This case was presented to the Brazilian Board of Orthodontics and Facial Orthopedics (BBO) as a requirement for the title of certified by the BBO.

## Abstract (pt)

A má oclusão de Classe I de Angle com biprotrusão é caracterizada pela inclinação acentuada dos incisivos para vestibular, o que provoca a protrusão dos lábios superior e inferior. As extrações dos primeiros pré-molares têm sido indicadas para reduzir a convexidade facial com a retração dos dentes anteriores, mantendo a relação de chave de oclusão dos caninos e dos primeiros molares. Para a obtenção de resultados compatíveis com as metas estéticas e cefalométricas idealizadas para o tratamento ortodôntico, é necessário que a fase de fechamento de espaços seja realizada com controle da sobremordida e do torque dos incisivos. Na maioria dos casos, também há necessidade de se proporcionar ancoragem máxima aos dentes posteriores. O presente caso clínico foi apresentado à Diretoria do Board Brasileiro de Ortodontia e Ortopedia Facial (BBO) como parte dos requisitos para a obtenção do título de Diplomado pelo BBO.

## INTRODUCTION

This paper reports the case of a 38-year and 6-month-old female patient who sought orthodontic treatment to improve facial esthetics and smile. Her medical history revealed no significant issues, except for allergy to iodine. Additionally, her clinical exams revealed that, even though the patient had good oral hygiene habits, she was susceptible to calculus formation - especially between mandibular incisors- and generalized gingival recession that included her upper central incisors. She had proper functional relationship between the tongue and the perioral muscles, associated with a nasal breathing pattern. Functional analysis of occlusion revealed that lateral guidance was performed by the canines on the right side, and by the first premolars on the left side. Anterior guidance was short, but did not completely disocclude the posterior teeth. There were no signs or symptoms of parafunctional habits or temporomandibular dysfunction.

## DIAGNOSIS

**Figure 1:**
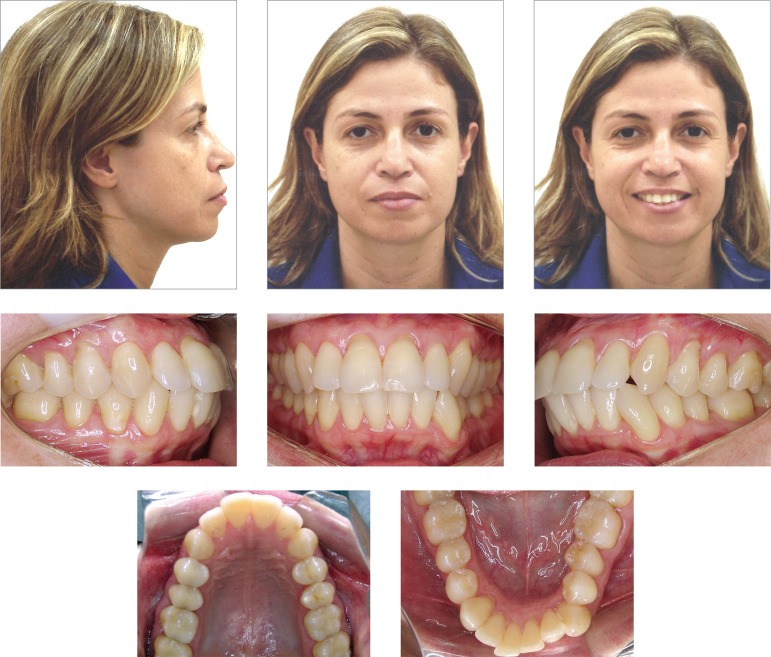
Initial facial and intraoral photographs.

The patient had a balanced facial pattern associated with convex profile and upper and lower lip protrusion (Ul-Line S = 2 mm; Ll-Line S = 1 mm), which provided her with decreased nasolabial angle and mentolabial sulcus, as well as increased nasolabial fold; however, without affecting passive lip sealing. At smiling, she clearly presented maxillary incisors protrusion and a wide buccal corridor (Fig 1).

**Figure 2:**
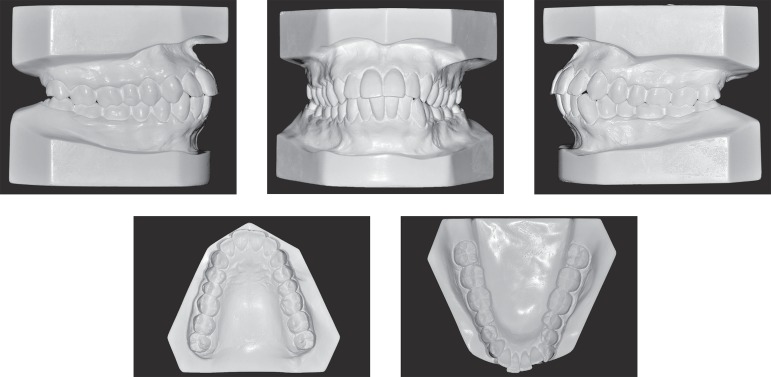
Initial casts.

Her dental analysis (Figs 1, 2) revealed Angle Class I malocclusion with 2 mm overjet and overbite. Teeth crowding was present in the lower arch with -6 mm discrepancy, infralabial inclination of tooth #41, linguoversion of tooth #32 and mesial infralabial inclination of tooth #33, which caused a 0.5-mm shift from the lower midline to the left. As for the upper dental arch, there was mild rotation of tooth #12. Both dental arches were severely elongated in the anteroposterior direction. Excess lingual tipping of lower premolars and molars caused transverse constriction.

**Figure 3:**
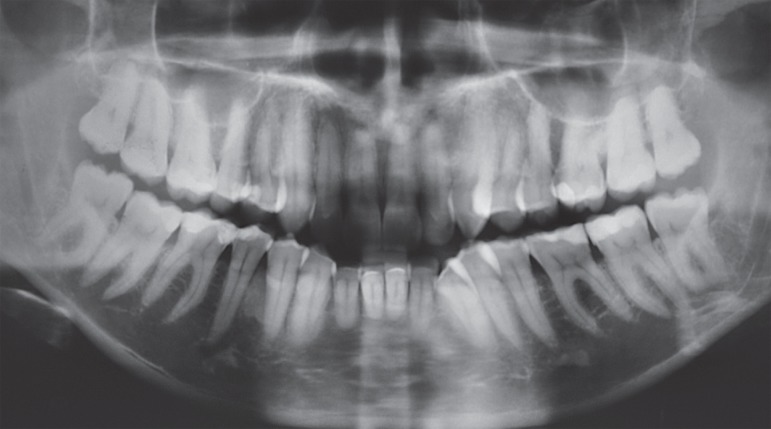
Initial panoramic radiograph.

**Figure 4:**
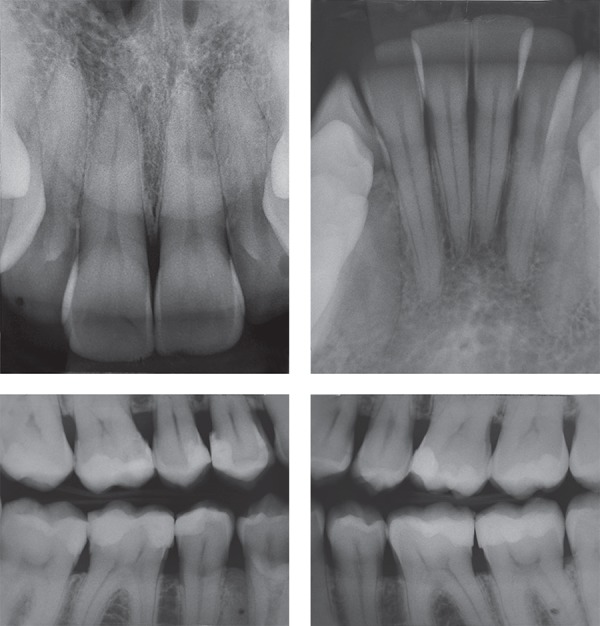
Initial periapical and interproximal radiographs of incisors.

Patient's panoramic radiograph revealed mesially tipped upper first molars (Fig 3). Periapical and interproximal radiographs of incisors revealed generalized reduction in alveolar bone crest height, which was more severe for the lower incisors. The exams also revealed excess restorative matter on the distal surface of tooth #14 (Fig 4).

**Figure 5:**
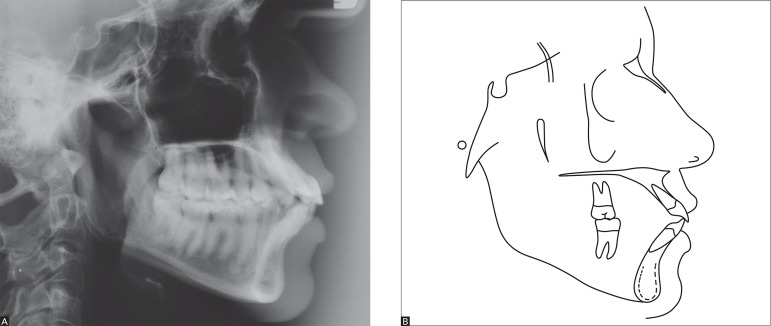
Initial lateral cephalogram (A) and cephalometric tracing (B).

Cephalometrically speaking (Fig 5, Tab 1), the patient had dentoalveolar bimaxillary protrusion associated with loss of Class II skeletal pattern caused by excess maxilla (SNA = 90.5º; SNB = 85º; ANB = 5.5º) and increased facial convexity (convexity angle = 14º). Nevertheless, these cephalometric values may have been influenced by severe protrusion (1-NA = 10 mm; 1-NB = 15 mm) and buccal tipping (1-NA = 32º; 1-NB = 51º; IMPA = 114º; 1/1 = 92º) of upper and lower incisors.

**Table 1:** Cephalometric measurements.

	Measurements		Normal	A	B	Dif. A/B
**Skeletal pattern**	SNA	(Steiner)	82°	90.5°	88.5°	2
	SNB	(Steiner)	80°	85°	87°	2
	ANB	(Steiner)	2°	5.5°	1.5°	4
	Angle of convexity	(Downs)	0°	14°	0.5°	13.5
	Axis Y	(Downs)	59°	59°	58.5°	0.5
	Facial angle	(Downs)	87°	91°	90°	1
	SN-GoGn	(Steiner)	32°	31°	27°	4
	FMA	(Tweed)	25°	24.5°	25°	0.5
**Dental pattern**	IMPA	(Tweed)	90°	114°	90°	24
	1 .NA (degrees)	(Steiner)	22°	32°	30°	2
	1 -NA (mm)	(Steiner)	4 mm	10 mm	7 mm	3
	1 .NB (degrees)	(Steiner)	25°	51°	26°	25
	1 -NB (mm)	(Steiner)	4 mm	15 mm	5.5 mm	9.5
	^1^⁄_1_ - Interincisal angle	(Downs)	130°	92°	125°	33
	1 -APo	(Ricketts)	1 mm	12 mm	4 mm	8
**Profile**	Upper lip — Line S	(Steiner)	0 mm	2 mm	-2 mm	4
	Lower lip — Line S	(Steiner)	0 mm	1 mm	-1 mm	2

## TREATMENT PLAN

Facial esthetics considered, treatment plan aimed at reducing lip protrusion, increasing nasolabial angle and softening the nasolabial fold. To make such alterations feasible, first upper and lower premolars extraction was recommended. According to the Visual Treatment Objective (VTO),^1^ a method that permits the analysis of tooth movement, the case was classified as in need for maximum anchorage. In other words, anterior teeth should be completely retracted towards the extraction spaces without allowing the posterior teeth to move mesially. To this end, mini-implants were indicated as an anchorage resource.^2^

Two other alternative treatment plans were considered. One of them included second premolars extraction, whereas the other included third molars extraction followed by distalization of dental arches with anchorage provided by miniplates. Nevertheless, neither possibilities were considered, since they required more complex and more invasive anchorage resources as well as longer treatment time.^3,4^

Initially, treatment plan included orthodontic appliance placed up to the second molars in both dental arches. Subsequently, first premolars extraction and mini-implant placement was performed on the four quadrants between second premolars and first molars, 8 mm away from the orthodontic arch on the attached gingiva and perpendicular to the buccal cortical bone.^5,6,7 ^Initially, mini-implants were used for partial canine retraction with active lacebacks^8^ gaining space for alignment of incisors and preventing their buccal inclination.

To close remaining extraction spaces, mass retraction of anterior teeth was performed by means of sliding mechanics^9 ^with active tie-backs hooked to mini-implants so as to produce an inclined force vector.

Case finishing was performed by repositioning of brackets followed by releveling and intercuspation with braided 0.019 x 0.025-in archwire and intermaxillary elastics. Retention consisted of a wraparound removable appliance for the upper arch, whereas for the lower arch a lingual arch bonded to the second premolars was recommended.

## TREATMENT PROGRESS

Orthodontic treatment was carried out using passive self-ligating pre-adjusted fixed appliances (MBT 0.022 x 0.028-in) on both arches, including second molars. Since the use of temporary orthodontic anchorage devices was not considered, tubes were bonded to the molars. Subsequently, first premolars extraction was requested and mini-implants were installed.

Alignment and leveling procedures began with NiTi SE 0.014-in wires followed by 0.016-in wire installed on both arches. During this period, canines, especially the lower ones, underwent distalization that provided space for incisors alignment and, as a result, prevented buccal tipping. Distalization was performed with active lacebacks placed from the mini-implants to the canine brackets. Lower lateral incisors were only included in the archwire after proper space was obtained. After initial alignment, anterior teeth remained tied together to prevent space opening. After the 0.016-in archwire was used, NiTi SE 0.014 and 0.016-in archwires were simultaneously installed. The association between arches aims at eliminating the slack established between leveling archwires and passive self-ligating brackets,^10 ^thus providing better rotational control and second-order expression. It is estimated that the association between 0.014 and 0.016-in archwires results in a total diameter of 0.021-in, which completely fills the brackets and tube slots.^11 ^Alignment and leveling were completed with the use of NiTi SE and stainless steel 0.019 x 0.025-in archwires. The former were distally bended to aid in the control of incisors buccal tipping, whereas the latter received hooks welded to the lateral incisors and canines, in addition to being diagramed and coordinated in accordance with the method advocated by Trevisi.^12^ During the visit booked for stainless steel archwire placement, the clinician also performed passive tie-backs with metallic 0.008-in ligatures from second molars to the hooks welded to the archwires. This procedure was performed to avoid proclination of anterior teeth with torque expression embeded in the brackets installed on these teeth.

Retraction of anterior teeth was performed by sliding mechanics carried out in association with mini-implants.

**Figure 6:**
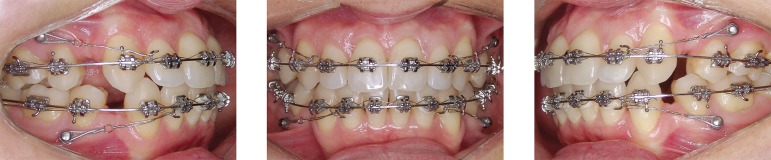
Progress of the anterior retraction phase.

Active tie-backs were installed from the mini-implants to the hooks welded to the archwires and activated 3 mm every 28 days^13^(Fig 6). The vertical position of mini-implants (placed approximately 8 mm from the orthodontic wires) was planned so as to produce an inclined force vector that, in association with sliding mechanics, would aid in achieving torque and overjet control during retraction of anterior teeth. This mechanics had a tendency towards distal tipping of first molars - more severe in the upper first molars - as a result of friction established between the tube and the orthodontic archwire. Such undesired movement was overcome in the subsequent phases of treatment.

Patient's intermediate records were performed in the final phase of anterior retraction after yielding the desired clinical outcomes. Based on patient's clinical and cephalometric exams, the clinician decided to remove the mini-implants and conclude residual space closure by mesially moving posterior teeth with the use of elastomeric chains, and keeping patient's anterior teeth tied together with the use of metallic ligature acting as anchorage unit.

Based on clinical as well as radiographic findings and after space closure, the brackets were repositioned to improve occlusal relationship. From this moment onwards, the teeth remained tied together in order to prevent space reopening. Treatment planning included intercuspation with 0.019 x 0.025-in braided archwires; however, due to the atypical anatomy of some teeth, treatment finishing included first and second-order bends performed in 0.016-in stainless steel archwires with the aid of 3/16-inch medium intermaxillary elastics in triangular disposition on canines and premolars. After assuring that esthetic, occlusal and functional outcomes were achieved, the orthodontic appliance was removed. The retention phase included an upper removable wraparound retainer used during the day and night within the first 6 months and during the night over the remaining period of time. In the mandibular arch, a 0.020-in stainless steel 5 x 5 intercanine fixed retainer with bypasses at the incisors and canines interproximal space was installed.

Treatment outcomes were achieved within the 27 months that comprised the active phase.

## RESULTS

**Figure 7:**
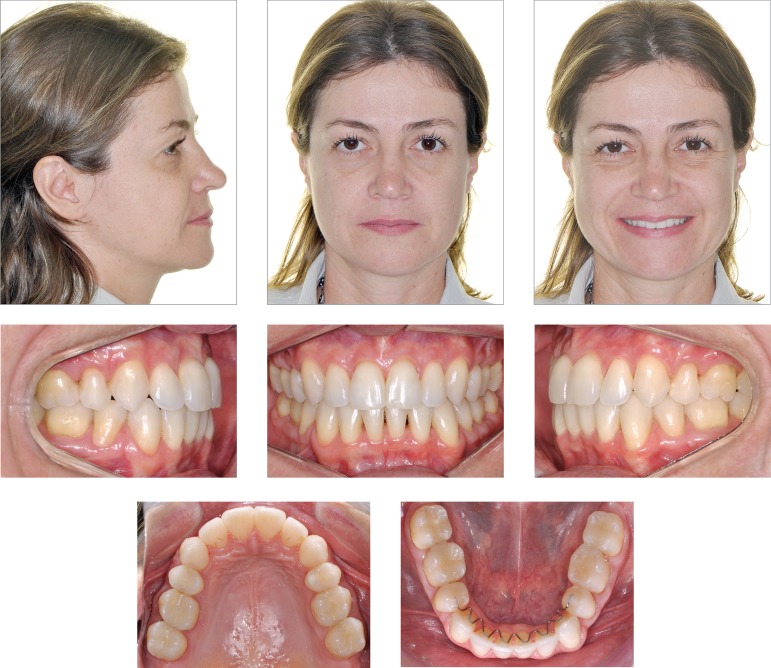
Final facial and intraoral photographs

Final treatment outcomes were considered highly satisfactory and met the objectives set at treatment onset. Patient's subjective facial analysis revealed a balanced facial profile with significant reduction in lip protrusion and nasolabial fold, as well as decreased incisors protrusion and buccal corridor at smiling (Fig 7). Additionally, upper and lower lips had a reduction of 4 mm and 2 mm, respectively, in relation to the S-line (Steiner), as shown in Fig 11 and Table 1.

**Figure 8:**
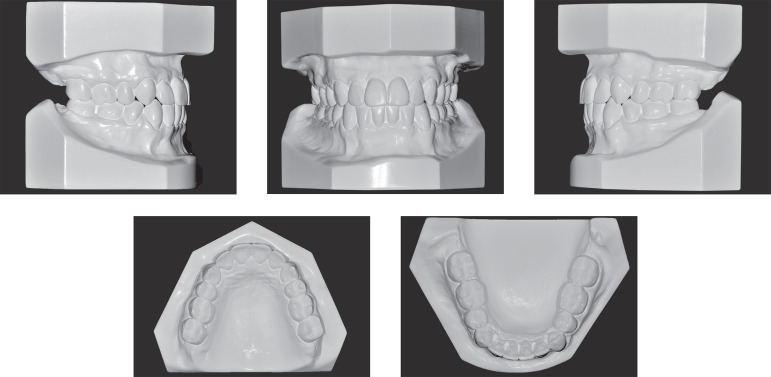
Final casts.

**Figure 9:**
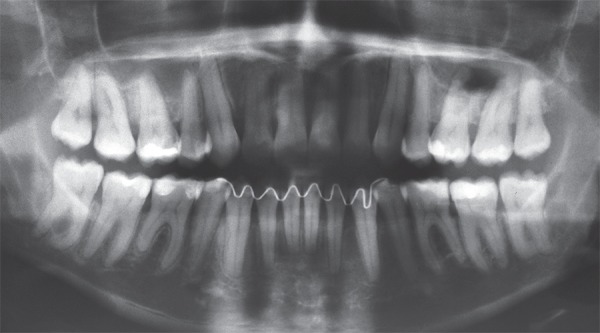
Final panoramic radiograph.

**Figure 10:**
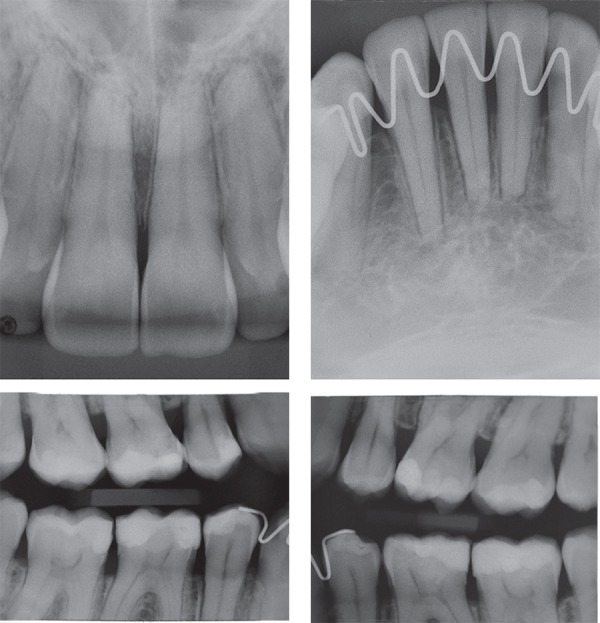
Final incisors periapical and interproximal radiographs.

**Figure 11:**
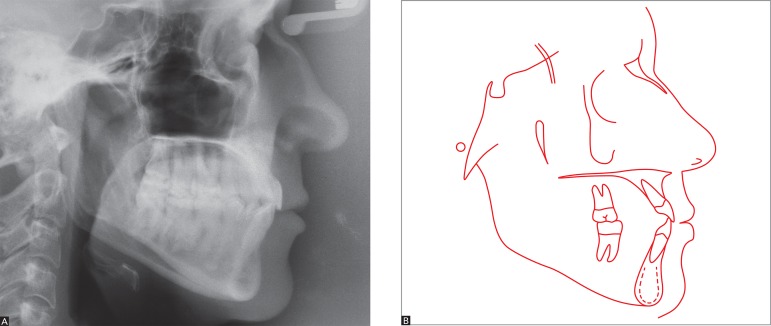
Final lateral cephalogram (A) and cephalometric tracing (B).

**Figure 12:**
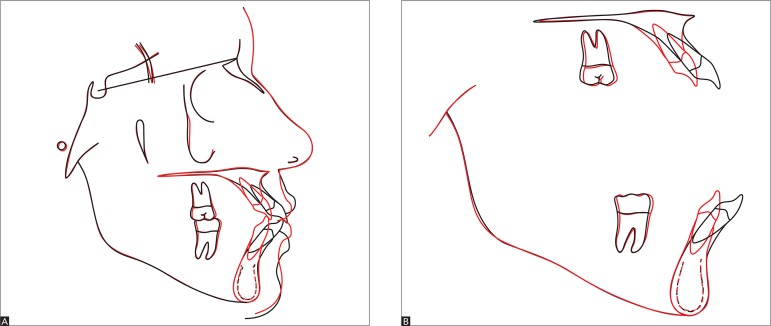
Initial (black) and final (red) cephalometric tracing total (A) and partial (B) superimposition.

In fact, the facial alterations occurred in response to movement applied to incisors. Upper incisors were retracted in 3 mm, with 2º of tipping in relation to the NA line; whereas the lower incisors were retracted in 9.5 mm, with 25ºof tipping in relation to the NB line. Additionally, there was a reduction of 24º in the IMPA angle. These changes caused an increase of 33ºin the interincisal angle (Figs 11, 12; Tab 1). It is worth noting that, despite achieving significant retraction of incisors, treatment outcomes revealed excellent overbite control (Figs 7, 8), which decisively contributed to restore esthetics and function. Cephalometric tracing superimposition revealed that tongue movement and intrusion of incisors occurred simultaneously (Fig 12). Such movement pattern (retraction and intrusion) also contributed to restore the periodontal health of incisors and to decrease gingival recession.

After initial alignment, black spaces emerged between lower incisors as a result of bone loss - previously observed at treatment onset - and triangular-shaped incisor crowns. During treatment finishing, interproximal enamel reduction was recommended to eliminate the black spaces. Nevertheless, this procedure could cause disproportional dental volume between the arches and, as a result, hinder overbite correction. Additionally, black spaces did not affect patient's smile esthetics and, for this reason, they were maintained instead of eliminated (Fig 7).

With regard to occlusion, case finishing was achieved with canines and first molars in key to occlusion and good intercuspation of posterior teeth (Figs 7 and 8). From a functional standpoint, proper adaptation of anterior overjet and overbite as well as torque control achieved for all teeth allowed incisal guidance in protrusion and lateral canine guidance to be obtained without any occlusal interference.

In general, treatment achieved good root parallelism, with distal angulation of canine roots and upper first molars, as revealed by the intermediate panoramic radiograph. Nevertheless, in the finishing phase, improvements in occlusal relationship were prioritized over correction of root angulation (Fig 9). Foreshortening of the distal root of tooth #46 and rounded incisors root, especially in the upper incisors (Figs 9, 10), were identified. This finding may be related to the association established between retraction and intrusion performed on those teeth.^14^ Additionally, panoramic radiographs (Fig 9) revealed that third molars remained unfavorably positioned. For this reason, they were extracted and are not shown in the final photographs and casts.

As expected, significant skeletal alterations did not occur. There was a reduction of 4º in the ANB angle and of 13.5º in the facial convexity angle (Fig 11, Tab 1) as a result of significant remodeling observed in the area of points A and B. Moreover, treatment with extractions provided significant improvements in patient's facial profile (Fig 12).

## FINAL CONSIDERATIONS

Bimaxillary protrusion is common among different ethnic groups. It is characterized by severe buccal tipping of anterior teeth and results in lip protrusion as well as increased facial convexity.^15^Conventional treatment includes extraction of first premolars to minimize facial convexity by retracting the anterior teeth and keeping canines and first molars in key to occlusion.^16,17 ^Drobocky and Smith^18 ^reported that 95% of patients treated with extraction of four first premolars have an average reduction of 3.4 mm and 3.6 mm in upper and lower lip protrusion in relation to the E line (Ricketts).

Nevertheless, although this treatment approach provides great predictability of results, treating bimaxillary protrusion by means of tooth extraction is a challenge for the orthodontist, especially during the space-closure phase. The main challenge is with regards to anchorage maintenance, since mesialization of posterior teeth may minimize retraction of anterior teeth and, as a result, hinder the esthetic and cephalometric objectives of orthodontic treatment. Particular attention should be given to torque control of incisors, since uncontrolled buccal tipping of incisors crown and, as a result, increased overbite may occur.^5^

Therefore, it is key that the orthodontist choose an efficient space closure method that also effectively controls potential side effects. After pre-adjusted fixed appliances were introduced, sliding mechanics has been considered as an alternative for space closure.^19 ^This method allows simultaneous retraction of incisors and canines. It is recommended that a 0.019 x 0.025-in stainless steel archwire be used in association with brackets and tubes with 0.022 x 0.028-in slots. Force may be produced by elastics or NiTi closed springs adapted between mini-implants and hooks welded to the archwire between canines and lateral incisors (Fig 6). In comparison to the use of archwires with loops, the sliding mechanics method is simpler, more esthetic and comfortable for the patient. Additionally, it produces lighter forces.^13,20^ Nevertheless, it is more sensitive in addition to being influenced by factors that hinder sliding of orthodontic archwires, such as friction, less torque control of anterior teeth and overbite.^21^Adding more torque on the orthodontic archwire and using brackets with greater torque are recommended to control tipping of incisors.^22^

With regard to anchorage control, there seems to be no significant difference between mass retraction of six anterior teeth and segmental retraction with initial distalization of canines followed by retraction of incisors.^23 ^In both methods, anchorage of posterior teeth is reinforced with conventional additional devices that, in certain situations, may be little effective or may strongly need patient's compliance. Such limitations are currently minimized with the use of mini-implants which have proved efficient in providing anchorage control and avoiding undesired mesial movement of molars, in addition to being widely accepted by patients.^24^Mini-implant placement require minimally invasive procedures and are reasonably affordable.

**Figure 13:**
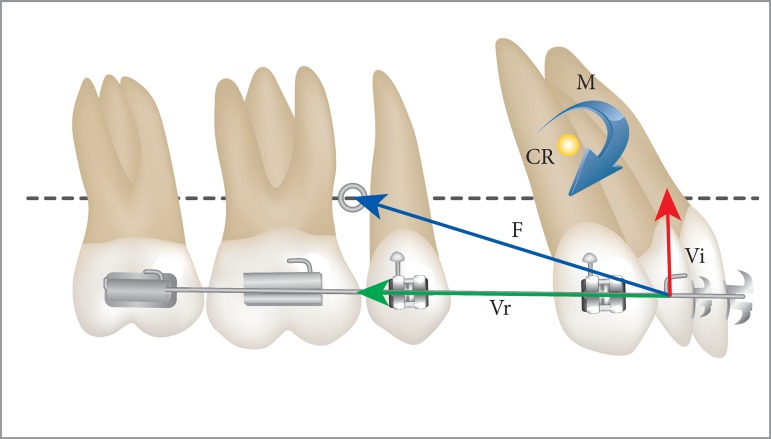
Schematic representation of forces produced by sliding mechanics associated with mini-implants: F = force produced by active tiebacks; Vr = horizontal retraction vector; Vi = vertical intrusion vector; M = incisors buccolingual tipping moment, with F going below the center of resistance (CR) of anterior teeth.

**Figure 14:**
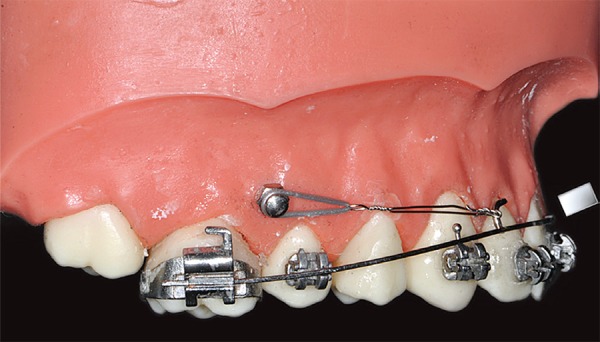
Effect of force applied to the orthodontic archwire during sliding mechanics associated with mini-implants.

Sliding biomechanics involved in mass retraction of anterior teeth aided by the use of mini-implants has some advantages that differ from other conventional methods of space closure. It is recommended that mini-implants used to aid sliding mechanics be placed between the roots of second premolar and first molar, 8 mm away from the orthodontic archwire.^5,6,7^ Mini-implants are apically placed in relation to the hook welded to the archwire and, for this reason, provide an inclined line of action of force that controls movement of anterior teeth. Decomposition of force results in a horizontal vector, responsible for retracting the anterior teeth; and a vertical vector, which contributes to achieve intrusion of these teeth during retraction and, as a consequence, allows effective overbite control. As the applied force goes below the center of resistance of anterior teeth,^25,26^it promotes movement that is likely to provide lingual tipping of these teeth (Fig 13). Nevertheless, should an inclined force be applied, it results in deflection of the orthodontic archwire, which simulates accentuation of upper the curve of Spee and favors torque control of anterior teeth (Fig 14). Such effect is of particular importance for passive self-ligating brackets, since they are less effective in the expression of torque.^27^ It is worth noting that deflection is extended to posterior teeth. As a result, it increases friction between the wire and the molar tubes, causing distal movement of these teeth.

Vertical positioning of mini-implants may vary according to the degree of the intrusion vector. Mini-implants more apically or cervically placed provide an intrusion vector of greater or lower degree that acts over anterior teeth. In order to avoid undesired tipping of the anterior occlusal plane, it is necessary that mini-implants be placed at the same height, taking both sides into account.

Additionally, it is worth noting that the use of mini-implants requires longer treatment time to provide complete space closure in comparison to the use of conventional resources. Such increase in treatment time may be caused by the movement produced by anterior teeth, given that posterior teeth do not contribute to minimize extraction spaces. More extensive movement of incisors may provide a higher risk of root resorption.^14^

The results of this study and the orthodontic literature led us to conclude that sliding mechanics associated with the use of mini-implants produces satisfactory effects for the treatment of bimaxillary protrusion with extraction of first premolars. Nevertheless, treatment success is primarily related to correct diagnosis and planning as well as to proper biomechanical principles applied to achieve the desired orthodontic movement.
